# “Segmental Necrotizing Granulomatous Neuritis”: A Rare Manifestation of Hansen Disease—Report of 2 Cases

**DOI:** 10.1155/2012/758093

**Published:** 2012-12-31

**Authors:** P. S. Jayalakshmy, P. H. Prasad, V. V. Kamala, R. Aswathy, Priya Pratap

**Affiliations:** ^1^Department of Pathology, Government Medical College, Thrissur, Kerala 680596, India; ^2^Department of Dermatology and Venereology, Government Medical College, Thrissur, Kerala 680596, India

## Abstract

Segmental necrotizing granulomatous neuritis (SNGN) is a rare condition affecting the nerves of Hansen disease patients. This is usually seen as a complication in association with the skin lesions of Hansen disease. Though very rare, it can also be the first presenting symptom of pure neuritic leprosy. We hereby report 2 cases of SNGN—one case of pure neuritic leprosy with initial presentation as SNGN and another, a treated case of borderline tuberculoid leprosy which relapsed with skin lesions and associated SNGN in the peripheral nerve.

## 1. Introduction 

Leprosy, the chronic infectious disease caused by *M. leprae* affects mainly the skin and the peripheral nerves. The disease is endemic in many tropical and subtropical countries but is declining in prevalence as a result of multidrug therapy and improved personal hygiene. The Indian subcontinent, Southeast Asia, sub-Saharan countries in Africa, and Brazil comprise the areas most affected at present [1].

Segmental necrotizing granulomatous neuritis (SNGN) is a rare complication seen in the peripheral nerves of Hansen disease patients. It can develop in association with a skin lesion or may be the initial presentation in pure neuritic leprosy. SNGN is usually associated with borderline tuberculoid leprosy. It can present as a single or multiple nodule/s of varying sizes along the course of a thickened peripheral nerve. In the earlier published literature, this lesion had been reported as nerve abscess. A patient presenting with a linear or nodular thickening of the peripheral nerve with impairment of sensation should always be investigated for Hansen disease even if there is no skin lesions. Early diagnosis of the condition and prompt treatment can cure the disease.

 Pure neuritic leprosy accounts for 4–8% of all leprosy cases [2]. The incidence of SNGN in pure neuritic leprosy has been estimated as 0.25% of all leprosy patients in the study by Chandi et al. [3]. In some cases, SNGN may be the only clinical manifestation of leprosy [4, 5]. Here, we are reporting 2 cases of segmental necrotizing granulomatous neuritis—one as the initial presentation of pure neuritic form of leprosy and the other as an associated peripheral nerve involvement in a treated and relapsed case of borderline tuberculoid leprosy with skin lesion.

## 2. Case History

### 2.1. Case  1

**Figure 1:**
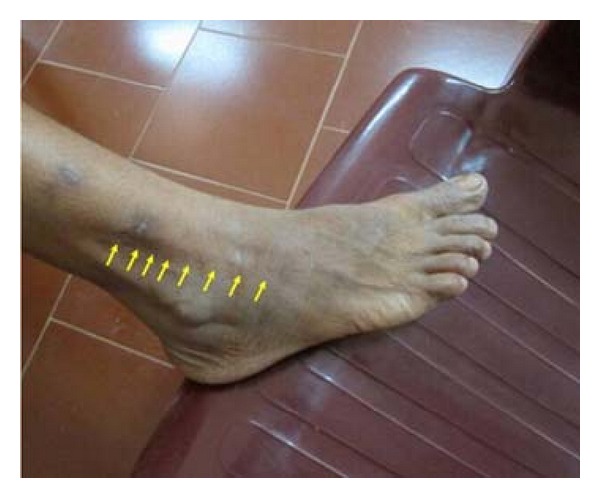
(Case  1): linear thickening of right superficial peroneal nerve.

30-year-old female presented with complaints of a linear cord like thickening in her right leg with numbness in the feet of 3-month duration. On examination, it was confirmed to be thickened superficial peroneal nerve with intermittent tiny nodular swellings (Figure 1). No other nerves were thickened. No skin lesions were seen. Nerve biopsy was done from an area of nodular thickening. 

**Figure 2:**
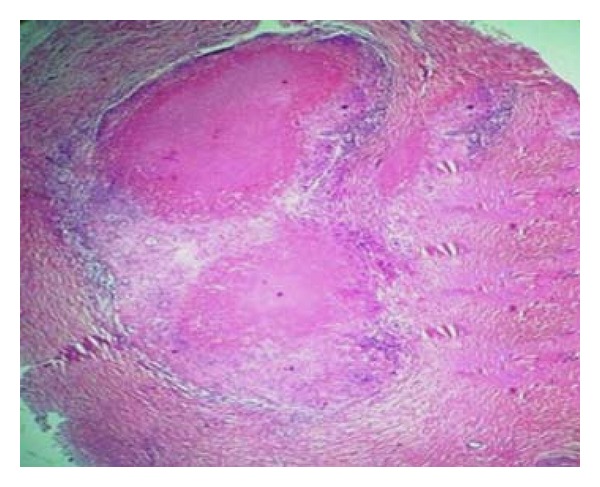
(Case  1): nerve biopsy, epithelioid granuloma with central caseous necrosis (H&E ×40).

**Figure 3:**
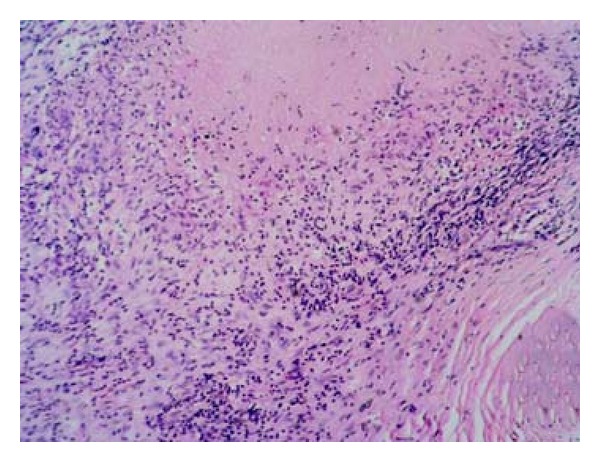
(Case  1): higher power view of Figure 2: Necrosis, Epithelioid cells and Lymphocytes seen (H&E ×100).

**Figure 4:**
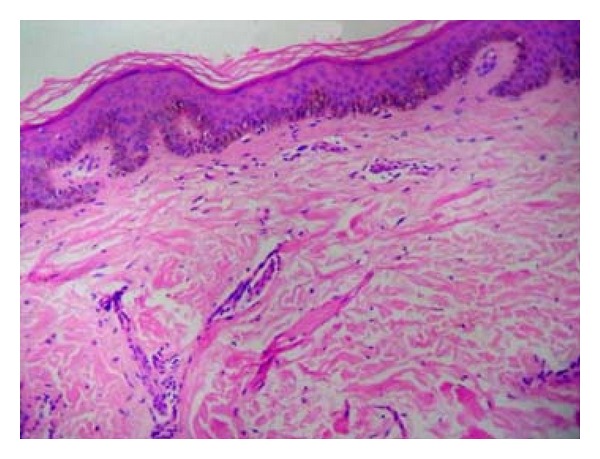
(Case  1): skin biopsy, perivascular lymphocytic infiltrate only. (H&E ×100).

 Histology of the nerve biopsy showed thickened nerve bundles showing multiple foci of caseous necrosis bordered by epithelioid cells and lymphocytes (Figures 2 and 3). The picture was typical of the entity segmental necrotizing granulomatous neuritis of leprosy (SNGN). Since there were no skin lesions, a random biopsy was taken from the skin near the thickened nerve which showed only mild nonspecific perivascular lymphocytic infiltrate (Figure 4) confirming that the patient was having pure neuritic leprosy.

### 2.2. Case  2

**Figure 5:**
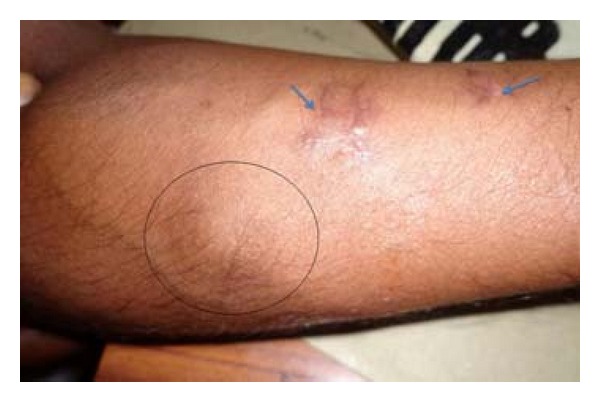
(Case  2): thickened left ulnar nerve with nodule (circled) and skin lesions (arrowed).

29-year-old male was diagnosed to have Hansen disease of borderline tuberculoid type 4-year back and was treated with multidrug regime. Now, the patient presented with hypopigmented, hypo aesthetic patches on the left forearm, and a soft nodular swelling of 3 × 2 cm size near the skin lesions along the course of the thickened left ulnar nerve for the last one month (Figure 5). Biopsy was taken from the skin lesion and the nodule in the affected nerve.

**Figure 6:**
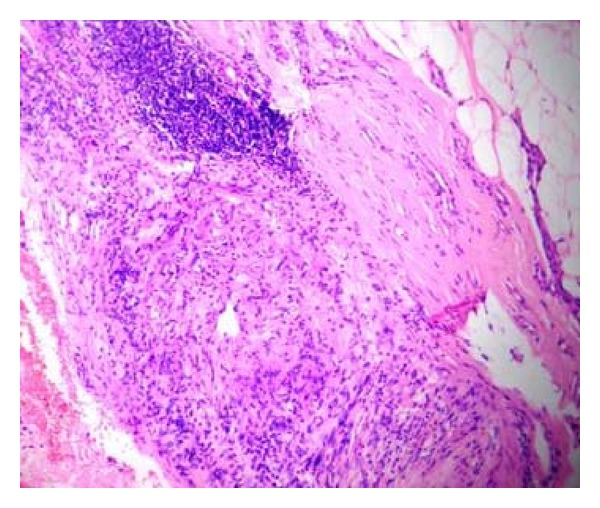
(Case  2): biopsy from skin lesion showing epithelioid granuloma in the subcutaneous nerve twig (H&E ×100).

**Figure 7:**
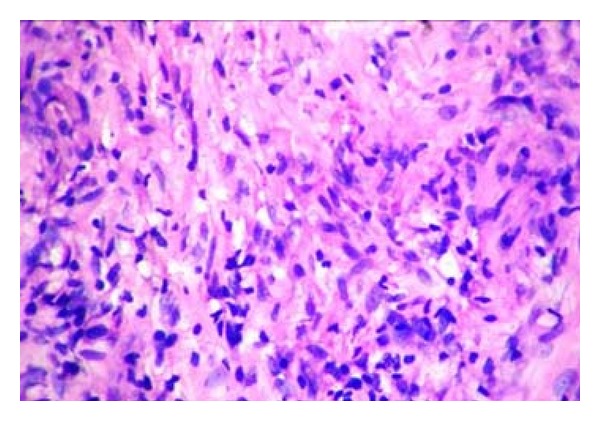
(Case  2): higher power view of epithelioid granuloma (H&E ×400).

**Figure 8:**
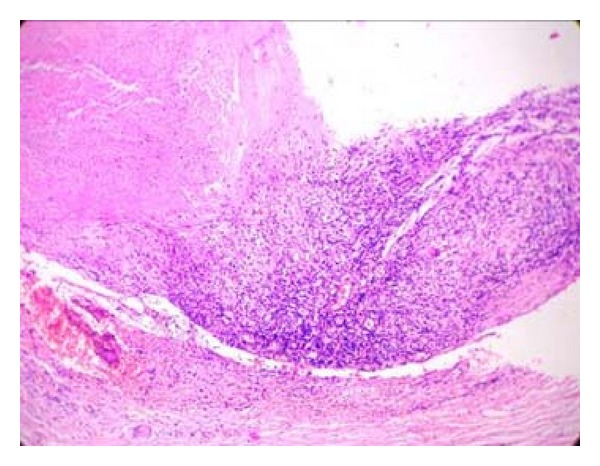
(Case  2): ulnar nerve biopsy showing caseating epithelioid granuloma (H&E ×40).

Microscopy of the skin lesion showed multiple epithelioid granulomas with sprinkling of lymphocytes mainly in the reticular dermis and in the subcutaneous nerve twigs (Figures 6 and 7). Histology of the nerve biopsy showed multiple epithelioid granulomas with central area of caseation necrosis (Figure 8). The case was diagnosed as borderline tuberculoid leprosy with SNGN in the nerve. Biopsies of both cases were negative for bacilli in acid-fast staining technique.

## 3. Discussion

The first publication about a nodular swelling in the peripheral nerve of a leprosy patient was by Muiri in 1924, which was described as “nerve abscess.” Since that, many case reports have been published about the condition as nerve abscess in leprosy.

Chandi et al. [3], in 1980, in their retrospective study of 30 cases, found necrotizing granulomatous inflammation of the nerves in some of these cases and first proposed for this condition the descriptive name-segmental necrotizing granulomatous neuritis of leprosy (SNGN) instead of nerve abscess. In this study, the authors stated that many of the cases reported, as nerve abscess previously, showed caseous necrosis surrounded by epithelioid cell granulomas and not a collection of neutrophils to describe it as an abscess, and the traditional collective name “nerve abscess” is therefore inappropriate [3]. Nerve abscess with neutrophil collection may be seen in type 2 reaction of leprosy. It should be distinguished from the specific entity SNGN, since in this condition epithelioid granulomas with caseous necrosis is seen and not neutrophil collection. 

SNGN is usually seen in association with borderline tuberculoid (BT) and Tuberculoid (TT) types of Hansen disease. In the study by Chandi et al. [3] SNGN with its characteristic focal caseous necrosis occurred more frequently in BT than in TT leprosy. Our first case was a pure neuritic leprosy with a borderline spectrum in the affected nerve. The second case was a borderline tuberculoid leprosy in the skin with involvement of the cutaneous nerve. A few organisms has been demonstrated in the lesions in different studies, but we could not detect the organism in both cases by the acid-fast staining technique. Since the SNGN lesion is seen in association with higher immunological status spectrum, the chance of detecting bacilli is very rare in routine special staining methods.

Type 1 reaction in leprosy usually is more prominent in the peripheral nerves than in the skin lesion. In the nerve, type 1 reaction induces increased intraneural inflammation and edema. The upgrading reaction results in caseous necrosis of large peripheral nerves [6]. Hence, SNGN may be considered as a form of type 1 upgrading reaction. 

The peripheral nerve trunk most prone to develop SNGN is the ulnar nerve just above the medial epicondyle of the humerus, with a greater predilection for the right ulnar nerve than the left [3]. In our second case, the ulnar nerve was the affected one, but the location of the lesion was in the left ulnar nerve in the forearm. In our first case, the nerve of the lower extremity—the superficial peroneal nerve—was involved. Isolated lesion of superficial peroneal nerve is very rare [7].

It is recognized that in BT and TT leprosy, caseation necrosis occurs in major nerve trunks as well as on occasion in the skin. Pure neuritic leprosy patients, though not having skin lesions, may show histological evidence of leprosy in biopsies from nasal mucosa and normal looking skin from hypoesthetic regions [8]. In our first case, the skin near the thickened nerve was biopsied which revealed only nonspecific perivascular lymphocytic infiltrate without any evidence of skin involvement, confirming the case as a pure neuritic leprosy. In patients with pure neuritic leprosy, subsequent skin lesions may develop. 

In our second case, skin lesion was the primary lesion and peripheral nerve lesion occurred later, and in the relapse stage, both cutaneous nerve and peripheral nerve showed SNGN.

## 4. Conclusion

Whenever a patient presents with nodular-thickened nerve and impairment of sensation, the patient should always be investigated for Hansen disease even if there is no skin lesions. Pure neuritic leprosy presenting as SNGN is very rare. Whenever epithelioid granulomas with caseation is seen in the peripheral nerve, Hansen disease should also be considered in the differential diagnosis. This nerve lesion is better diagnosed as SNGN and not as a nerve abscess, as previously described to avoid confusion with a true abscess. Early diagnosis of the condition and prompt treatment can cure the disease before the occurrence of mutilating complications.
